# Medication economic burden of antidepressant non-adherence in Spain

**DOI:** 10.3389/fphar.2023.1266034

**Published:** 2023-11-14

**Authors:** Eduardo Gutiérrez-Abejón, M. Aránzazu Pedrosa-Naudín, Diego Fernández-Lázaro, F. Javier Alvarez

**Affiliations:** ^1^ Pharmacological Big Data Laboratory, Department of Cell Biology, Genetics, Histology and Pharmacology, Faculty of Medicine, University of Valladolid, Valladolid, Spain; ^2^ Valladolid Este Primary Care Department, Valladolid, Spain; ^3^ Pharmacy Directorate, Castilla y León Health Council, Valladolid, Spain; ^4^ Facultad de Empresa y Comunicación, Universidad Internacional de la Rioja (UNIR), Logroño, Spain; ^5^ Department of Cellular Biology, Genetics, Histology and Pharmacology, Faculty of Health Sciences, Campus of Soria, University of Valladolid, Soria, Spain; ^6^ Neurobiology Research Group, Faculty of Medicine, University of Valladolid, Valladolid, Spain; ^7^ CEIm, Hospital Clínico Universitario de Valladolid, Valladolid, Spain

**Keywords:** antidepressant, medication adherence, drug utilization, depression, anxiety, mental disorders, psychotropic drugs

## Abstract

**Introduction:** Non-adherence to antidepressants is associated with worse disease outcomes (morbidity and mortality) and correlates with higher healthcare resource utilization and costs.

**Methods:** A population-based registry study was conducted to assess non-adherence and to analyze the economic burden of treatment and from non-adherence to antidepressants in 2021. Non-adherence was measured by the Medication Possession Ratio and those below 80% were classified as non-adherent.

**Results:** In 2021, 246,718 patients (10.60% [95% CI: 10.48–10.72]) received antidepressants at a cost of €29 million. The median antidepressant cost per patient/year was €70.08€, ranging from €7.58 for amitriptyline to €396.66 for agomelatine. Out-of-pocket costs represented 6.09% of total expenditures, with a median copayment of €2.78 per patient. The 19.87% [95% CI 19.52–20.22)] of patients were non-adherent to antidepressants, costing €3.9 million (13.30% of total antidepressant costs). Non-adherence rates exceeded 20% for the tricyclic antidepressants, fluoxetine (23.53%), fluvoxamine (22.42%), and vortioxetine (20.58%). Venlafaxine (14.64%) and citalopram (14.88%) had the lowest non-adherence rates, of less than 15%. The median cost of non-adherent medications per patient/year was €18.96 and ranged from €2.50 (amitriptyline) to €133.42 (agomelatine).

**Conclusion:** Reducing non-adherence to antidepressants is critical to improving clinical and economic outcomes. The implementation of interventions and standardized measures, including early detection indicators, is urgently needed. Antidepressants differ with regard to non-adherence and their cost, and this should be considered when prescribing this medication. The Medication Possession Ratio could be used by the healthcare provider and clinician to identify non-adherent patients for monitoring, and to take necessary corrective actions.

## 1 Introduction

Depression is the most common mental health disorder, affecting more than 300 million people worldwide (GBD, 2019 Mental Disorders Collaborators, 2022). Depressive disorders are associated with high economic costs to society and healthcare systems; in particular, major depressive disorder accounted for $326.2 billion in global expenditures in the United States in 2018 (Greenberg et al., 2021). In addition, pharmacy expenditures were $20.4 billion, representing 6% of direct costs (Greenberg et al., 2021), much higher lees than a decade ago. Based on European labeling, antidepressants are also used in the treatment of anxiety, insomnia, eating disorders and pain of neuropathic origin, among others.

Non-adherence is an important precipitating factor in the loss of efficacy of antidepressants. Non-adherence to antidepressants is not only associated with worse disease outcomes (morbidity and mortality), but is also strongly correlated with healthcare resource utilization and costs (Ho et al., 2016). Non-adherence to antidepressants is a major problem worldwide, estimated at up to 50% (WHO, 2003; Wong et al., 2022). In Spain, 10.6% of the population was reported to be taking antidepressants in 2021and 19.9% of the patients were non-adherent (Pedrosa-Naudín et al., 2022). Information on the costs of antidepressant non-adherence is lacking. A real-world population-based registry study was conducted to assess antidepressant non-adherence, pharmacy costs of antidepressants, and pharmacy costs of non-adherent patients to antidepressants in the free public national health system in Castille and Leon (Spain) in 2021. This study aimed to describe i) antidepressant non-adherence, ii) medication costs of antidepressant use, and iii) medication costs of those non-adherents to antidepressants. This is analyzed for each of the 26 antidepressants available in Spain.

## 2 Methods

### 2.1 Real-world study details

We conducted a population-based economic evaluation of antidepressant use in Castile and Leon, Spain, in 2021, covering a population of 2,327,420. We followed the CHEERS (Husereau et al., 2022) reporting guideline.

The focus was on patients who used at least one antidepressant in 2021. Data were collected from CONCYLIA (http://www.saludcastillayleon.es/portalmedicamento/es/indicadores-informes/concylia, accessed on 5 May 2023), the pharmacy information system for Castile and Leon (Spain). CONCYLIA integrates information on medicines dispensed in pharmacies and reimbursed by the Spanish National Health System. It also includes prescription records and is linked to the electronic prescription system in Castile and Leon (Spain). The data are anonymized using the patient identification code, which also allows the retrieval of sociodemographic and health data, including diagnoses. However, data on hospital care and private prescriptions are not available in CONCYLIA. The database is considered to be highly valid and reliable due to the integration of prescription and dispensing data (Pedrosa-Naudín et al., 2022). In this study, all dispensations were considered equivalent to consumption, following the approach used previously (Gutiérrez-Abejón et al., 2017; 2020; Pedrosa-Naudín et al., 2022). In the region of Castile and Leon (Spain), the typical duration of antidepressant treatment is usually between 6 months and a year (Gutiérrez-Abejón et al., 2020).

Access to the information was granted by the Castile and Leon Health Council Pharmacy Directorate, and the study protocol was approved by the East Valladolid Area Ethics Committee on 24 February 2022 (reference number PI 22-2622).

### 2.2 Variables

Non-adherence to antidepressants was determined using the medication possession ratio (MPR) (Pedrosa-Naudín et al., 2022). MPR is a method based on pharmacy records that indirectly measures medication adherence. It was calculated for each patient by dividing the days of supply during a specific monitoring period (1 year) by the number of days from the first dispensing to the end of the monitoring period (Andrade et al., 2006). MPR was determined based on prescribing and dispensing records for each patient and for each type of antidepressant used. MPR is expressed as a percentage and considered a continuous variable. Patients with an MPR below 80% were categorized as non-adherent, which is a commonly accepted cut-off point in the literature (Baumgartner et al., 2018; Liu et al., 2022).

Other variables collected in the study included sociodemographic information, such as age and gender, and health-related variables, such as diagnoses.

Diagnoses were categorized into different groups based on the International Classification of Diseases-10 (ICD-10) criteria. These groups included depression, anxiety, depression and anxiety, pain of various causes (neuropathic pain, cephalalgia, and migraine), and other psychiatric disorders. The categorization was done according to the approved indications of the relevant drugs, following the European labeling guidelines (EMA, 2022). Twenty-six antidepressants available in Spain were included in the analysis, identified using the ATC (Anatomical Therapeutic Chemical) classification system (WHO Collaborating Centre for Drug Statistics Methodology, 2020). Antidepressant dispensing was measured by the number of packages and the defined daily doses (DDDs) for each medication.

Medication expenditures were calculated using the prices listed in the official medicines formulary of the Spanish National Health System (https://www.sanidad.gob.es/profesionales/nomenclator.do, accessed on 5 May 2023). In terms of cost/DDD, the most expensive antidepressants were agomelatine (€1.25/DDD), reboxetine (€1.20/DDD), and vortioxetine (€1.10/DDD). On the other hand, the cheapest antidepressants were fluoxetine (€0.09/DDD), amitriptyline (€0.17/DDD) and imipramine (€0.21/DDD) (Table 1). All costs were reported in euros using 2021 values. The exchange rate of €1 = US$1.1326 was based on the European Central Bank rate on 31 December 2021.

**TABLE 1:** Antidepressants Non-adherence and cost in Castille and Leon (Spain) in 2021. Data is presented according to the highest to the lowest cost of antidepressants.

Antidepressants	Patients, No.	Cost/DDD, €	Total costs, €	Cost per patient, € median (interquartile range)	Non-adherence prevalence (95% CI, confidence interval	Non-adherence costs, €	Non-adherence cost per patient, € median (interquartile range)
Escitalopram	39,740	0.31	4613167.21	96.36 (43.79–131.35)	18.78 (18.56–19.01)	621193.87	35.04 (17.52–78.84)
Duloxetine	16,865	0.95	4334212.26	173.68 (80.19–347.36)	17.41 (17.19–17.63)	573416.82	66.81 (26.72–187.11)
Mirtazapine	32,428	0.57	3724260.41	85.25 (34.10–119.35)	16.53 (16.31–16.74)	418127.44	25.56 (17.05–68.19)
Venlafaxine	19,923	0.42	3467997.48	115.08 (71.87–229.80)	14.64 (14.43–14.84)	379540.45	57.54 (19.15–134.05)
Desvenlafaxine	9,742	0.73	3270190.95	278.04 (115.85–444.72)	18.56 (18.34–18.78)	449477.72	111.18 (37.06–268.70)
Sertraline	29,579	0.19	2420658.04	64.14 (23.36–93.28)	18.76 (18.53–18.98)	347164.21	23.32 (11.66–58.30)
Vortioxetine	6,019	1.10	2026639.85	204.60 (68.20–432.97)	20.58 (20.35–20.82)	306840.1	71.10 (34.09–212.83)
Paroxetine	27,160	0.22	1755085.06	60.90 (30.45–73.08)	19.32 (19.09–19.54)	257652.02	24.36 (12.18–48.72)
Trazodone	26,326	0.32	777488.46	19.00 (9.49–37.92)	17.79 (17.57–18.01)	102465.04	6.32 (6.32–18.96)
Bupropion	3,564	0.99	663601.34	130.05 (48.75–195.00)	21.27 (21.03–21.5)	100817.44	48.75 (16.25–113.75)
Citalopram	9,317	0.18	453214.72	35.84 (20.48–61.44)	14.88 (14.67–15.08)	49630.72	15.36 (7.68–38.40)
Agomelatine	1,062	1.25	452554.04	396.66 (144.24–468.78)	17.89 (17.67–18.11)	64882.38	133.42 (61.30–367.80)
Fluoxetine	12,603	0.09	379611.65	26.25 (10.50–31.90)	23.53 (23.29–23.78)	71984.7	12.50 (5.25–25.90)
Amitryptiline	20,971	0.17	316825.22	7.58 (2.50–14.84)	23.46 (22.93–23.99)	49300.44	2.50 (2.12–6.36)
Clomipramine	2,831	0.36	257872.01	60.32 (26.64–105.56)	23.31 (22.78–23.84)	46749.62	35.89 (12.48–82.94)
Tianeptine	557	0.75	85109.28	112.40 (44.96–179.84)	19.75 (19.52–19.98)	12341.52	44.96 (22.48–112.40)
Reboxetine	412	1.20	82720.44	179.30 (83.72–215.16)	17.72 (17.5–17.94)	12001.66	71.74 (35.86–179.30)
Fluvoxamine	660	0.26	50488.32	49.92 (30.72–92.16)	22.42 (22.19–22.66)	8889.6	30.72 (7.68–61.44)
Mianserin	624	0.39	24364.84	17.68 (5.64–35.82)	21.96 (21.72–22.19)	3665.14	5.64 (4.42–16.92)
Maprotiline	346	0.31	15,393.9	19.14 (12.00–44.66)	23.99 (23.45–24.52)	2754.82	6.38 (6.38–25.52)
Nortiptyline	201	0.23	5527.24	16.74 (5.78–29.68)	24.38 (23.84–24.92)	1042.38	6.18 (2.06–21.20)
Doxepine	86	0.20	1891.84	10.51 (4.09–18.64)	15.12 (14.67–15.56)	214.09	4.09 (2.33–5.54)
Imipramine	46	0.21	1081.01	12.66 (6.02–24.70)	52.17 (51.55–52.8)	465.89	11.14 (3.74–21.07)
Moclobemide	14	0.35	1025.14	67.34 (62.16–103.50)	28.57 (4.91–52.24)	191.61	46.61 (25.88–69.93)
**TOTAL**	246,718	0.39	29180980.71	70.08 (25.57–151.16)	19.87 (19.71–20.03)	3880809.68	18.96 (8.52–54.81)

Abbreviation: DDD, defined daily dose.

To convert € to US, dollars, multiply by 1.13.

### 2.3 Statistical analysis

Results are presented as percentages with 95% confidence intervals (95% CI) or medians with interquartile ranges (IQR). Differences between groups were assessed using the Mann-Whitney test and Kruskal–Wallis test for continuous variables, as appropriate. Statistical analyses were performed with SPSS version 24.0 (SPSS Inc, Chicago, IL). A significance level of *p* ≤ 0.05 was used to determine statistical significance.

## 3 Results

In 2021, 246,718 patients (10.60% [95% CI: 10.48–10.72]) received antidepressants at a cost of €29 million. The five most prescribed drugs were escitalopram, mirtazapine, sertraline, paroxetine, and trazodone, accounting for 63.49% of prescriptions. The combined cost of escitalopram, duloxetine, mirtazapine, venlafaxine, and desvenlafaxine accounted for 66.51% of expenditures. The median cost of antidepressants per patient/year was €70.08€, ranging from €7.58 for amitriptyline to €396.66 for agomelatine (Table 1). Out-of-pocket costs accounted for 6.09% (€1,775,910) of total expenditures, with a median copayment of €2.78 95% CI: (0.58–9.00) per patient. A total of 49,024 patients (19.87% [95% CI 19.52–20.22)] were non-adherent to antidepressants, costing €3.9 million (13.30% of total antidepressant costs, Table 1). Nonadherence rates exceeded 20% for tricyclic antidepressants and for fluoxetine (23.53%), fluvoxamine (22.42%), and vortioxetine (20.58%). Venlafaxine (14.64%) and citalopram (14.88%) had the lowest non-adherence rates, below 15%, and minimal impact on total costs (10.94% and 10.95%, respectively). The median cost of non-adherent medications per patient/year was €18.96 and ranged from €2.50 (amitriptyline) to €133.42 (agomelatine) (Table 1).

Male patients (€70.49), those aged 65–79 years (€76.72), and those with a mixed diagnosis of depression and anxiety (€105.10) had the highest medication costs per patient. The costs of non-adherent patients showed the same pattern (Table 2).

**TABLE 2:** Antidepressant cost per patient analyzed by gender, age range, and mental health diagnosis.

	Patients No.	Cost per patient € median (interquartile range)	Patients No.	Non-adherence cost per patient € median (interquartile range)
Gender				
*Male*	71,340	68.18 (23.32–146.96)	12,160	57.54 (24.36–122.64)
*Female*	175,378	70.49 (26.27–151.58)	29,649	52.56 (24.36–113.88)
	p	0.001		0.001
Age range				
*<25*	6368	27.10 (9.78–70.08)	1462	35.04 (15.50–78.84)
*25–64*	114,512	68.16 (23.04–157.56)	23,854	56.96 (24.36–122.64)
*65–79*	63,702	76.72 (31.50–162.00)	8854	58.30 (26.26–119.19)
*≥80*	62,136	70.08 (30.00–136.40)	7639	48.72 (23.04–102.30)
	p	0.001		0.001
Mental health diagnostics				
*Depression*	90,629	73.08 (29.70–166.22)	13,230	60.90 (26.72–128.56)
*Anxiety*	72,130	68.25 (27.50–131.34)	13,534	52.56 (26.28–106.88)
*Depression and Anxiety*	54,435	105.10 (51.15–210.24)	9888	78.80 (37.75–160.32)
*Pain (different etiologies)*	24,773	14.71 (4.46–68.01)	4720	13.78 (6.36–53.45)
*Other psychiatric diagnoses*	19,595	76.68 (32.50–170.69)	3193	60.90 (26.28–129.96)
	p	0.001		0.001

To convert € to US, dollars, multiply by 1.13.

## 4 Discussion

The 10.6% of the population was taking antidepressants and 19.87% of the patients were non-adherent. Urban residence, male sex, use of tricyclic antidepressants, and a diagnosis of neuropathic pain were underlying predictors of medication non-adherence, as we have previously reported (Pedrosa-Naudín et al., 2022). In the current study, we show results for each of the 26 antidepressants available in Spain.

The use of antidepressants is high and has been increasing for several years (COVID-19 Mental Disorders Collaborators, 2021). The pattern of antidepressant use and gender distribution in our study was consistent with other manuscripts (Cutler et al., 2018; COVID-19 Mental Disorders Collaborators, 2021). The most used antidepressant was escitalopram, ahead of citalopram, which has been the most used in recent years (Lalji et al., 2021). This is logical because escitalopram has been shown to be the most effective of the SSRIs (Khoo et al., 2015). However, escitalopram has been reported to be associated with higher costs and higher rates of nonadherence than citalopram. In addition, escitalopram has been surpassed in efficacy by other antidepressants such as mirtazapine, venlafaxine, and agomelatine. In this case, agomelatine will not be cost-effective (Khoo et al., 2015) because its cost/DDD is the highest of all antidepressants. As a result, the use of agomelatine has been reduced.

Antidepressant non-adherence was lower than previously reported in other countries (WHO, 2003; Kales et al., 2016; Wong et al., 2022) and in Spain (Párraga Martínez et al., 2014; Baeza-Velasco et al., 2019). Nevertheless, comparisons should be made with caution due to methodological differences, particularly in the measurement of adherence. In addition to citalopram, venlafaxine and mirtazapine were the antidepressants associated with lower rates of non-adherence, because these drugs improve situations that promote non-adherence, such as reduced cognitive and sexual function or weight gain (Lalji et al., 2021). The total cost of antidepressant was €29 million, and non-adherence to antidepressants accounted for 13.30% of the total cost, representing a significant economic burden for the Spanish national health system. To our knowledge, this is the first study in our country to measure the economic impact of medication non-adherence on drug expenditures. While most research has focused on the increase in healthcare services utilization and associated costs due to non-adherence, this study shows that the cost of discontinued antidepressant is not insignificant. The median cost of antidepressant medication was of €70.08 per patient/year, which is significantly lower than that reported in the United States (Greenberg et al., 2021), and in other European countries, as Germany (Jacob and Kostev, 2016). One factor contributing to non-adherence is out-of-pocket costs (Luiza et al., 2015; Sinnott et al., 2016; González López-Valcárcel et al., 2017), particularly for high-cost medications (Schneeweiss et al., 2007). In a study conducted in our country (González López-Valcárcel et al., 2017), a reduction in adherence of 6.8%–8.3% was observed for medications such as statins, angiotensin-converting enzyme inhibitors (ACEI), and angiotensin II receptor blockers (ARB). This decrease occurred primarily among older, retired patients and those with lower incomes. On the other hand, the researchers observed that the co-payment increase had no significant effect on lower-priced drugs such as antiplatelet agents and beta-blockers (González López-Valcárcel et al., 2017).

Conflicting results have been observed with antidepressants. Co-payments of between €0.50 and €1.50 per package have been shown to promote non-adherence more for antidepressants than for other medications (Sinnott et al., 2016). However, other studies found no differences with other medications (Goldman et al., 2004) or only in women (Ong et al., 2003). In contrast, one study found that a €1 increase in co-payment did not affect antidepressant non-adherence (Linnet et al., 2013).

In Spain, chronic medications such as antidepressants are subject to a co-payment of between 0% and 10%, up to a maximum of €4.24 per package (Spanish Ministry of Health, 2015). In our study, out-of-pocket costs accounted for 6% of total antidepressant expenditures, with a median value of €2.78/patient/year. However, a high percentage of non-adherence was observed for fluoxetine, the medicine with the lowest cost/DDD of all antidepressants. The study was not without several limitations. An indirect method based on MPR was used, considering filling as a proxy for medication taking. A cut-off of 80% in the MPR was used, but it is not a universal score (Baumgartner et al., 2018). However, MPR may overestimate adherence compared with more conservative measures such as the proportion of days covered (PDC) (Gelzer et al., 2019). In this sense, PDC provides a more accurate measure of medication adherence and has been recommended by several institutions, including the Pharmacy Quality Alliance, to measure adherence in polymedicated patients (Canfield et al., 2019). Despite significant limitations, MPR is widely used to measure adherence in chronic diseases because of its ease of calculation and low cost (Boulet et al., 2012). Furthermore, data on antidepressant use in hospital and private prescriptions are not accessible through our data source, CONCYLIA. However, due to the sample size and the fact that antidepressants covered are prescription medicines, the biases introduced by the use of dispensing data are not considered relevant (Pedrosa-Naudín et al., 2022) within the free public health system in Spain. In addition, caution should be exercised when comparing figures from other countries, especially outside the European Union, because of possible differences in the costs of antidepressant medication. This real-world study provides a comprehensive view of the population covered by the free Spanish National public health system. In conclusion, escitalopram was the most used antidepressant, while citalopram was associated with the lowest rate of non-adherence. In addition, fluoxetine was the least expensive antidepressant and, surprisingly, the one with the highest percentage of costs associated with non-adherent patients. Antidepressants differ about the non-adherence and their cost, and this should be considered when prescribing this medication.

Reducing non-adherence to antidepressants is critical to improving clinical and economic outcomes (Ho et al., 2016). Thus, there is an urgent need to implement strategies such as the integration of measures for the early detection of non-adherence. MPR could serve as a tool for healthcare providers and clinicians to identify non-adherent patients for close monitoring and intervention as needed.

Lastly, in this sense, previous research conducted by our group concluded that MPR should serve as the physician’s preferred indicator of antidepressant non-adherence (Pedrosa-Naudín et al., 2022). However, the role of the pharmacist as a key figure in patient care should not be ignored (Lallemand et al., 2023). Spain has an extensive network of more than 22,000 community pharmacies (Amador-Fernández et al., 2023), making it easier for pharmacists to build trusting relationships with their patients (Pousinho et al., 2016). As part of their pharmaceutical care, community pharmacists should provide patients with personalized strategies to achieve optimal adherence to prescribed medications (Jairoun et al., 2023). In fact, there are numerous examples in the literature of successful adherence improvement through community pharmacy initiatives (Nogueira et al., 2020; Jairoun et al., 2023; Lallemand et al., 2023; Sáez-Benito et al., 2023).

## Data Availability

The data analyzed in this study is subject to the following licenses/restrictions: Restrictions apply to the availability of these data. Data were obtained from regional health authorities (Gerencia Regional de Salud (GRS)) and may be requested from conciertofco@saludcastillayleon.es. Requests to access these datasets should be directed to conciertofco@saludcastillayleon.es.
